# Usefulness of the velocity–time integral of the left ventricular outflow tract variability index to predict fluid responsiveness in patients undergoing cardiac surgery

**DOI:** 10.1186/s44156-023-00022-z

**Published:** 2023-06-29

**Authors:** Aldo Pérez-Manjarrez, Edgar García-Cruz, Rodrigo Gopar-Nieto, Gian Manuel Jiménez-Rodríguez, Emmanuel Lazcano-Díaz, Gustavo Rojas-Velasco, Daniel Manzur-Sandoval

**Affiliations:** 1 Cardiovascular Critical Care Unit, Instituto Nacional de Cardiología Ignacio Chávez, Juan Badiano 1, Belisario Domínguez, Sección XVI, Tlalpan, P.O. Box 14080, Mexico City, Mexico; 2 Adult Congenital Heart Disease Unit, Instituto Nacional de Cardiología Ignacio Chávez, Mexico City, Mexico; 3 Coronary Care Unit, Instituto Nacional de Cardiología Ignacio Chávez, Mexico City, Mexico

**Keywords:** Fluid responsiveness, Cardiac surgery, Hemodynamic monitoring

## Abstract

**Background:**

Haemodynamic monitoring of patients after cardiac surgery using echocardiographic evaluation of fluid responsiveness is both challenging and increasingly popular. We evaluated fluid responsiveness in the first hours after surgery by determining the variability of the velocity–time integral of the left ventricular outflow tract (VTI-LVOT).

**Methods:**

We conducted a cross-sectional study of 50 consecutive adult patients who underwent cardiac surgery and in whom it was possible to obtain VTI-LVOT measurements. We then determined the variability and correlations with our pulse pressure variation (PPV) measurements to predict fluid responsiveness.

**Results:**

A strong positive correlation was seen between the VTI-LVOT variability index absolute values and PPV for predicting fluid responsiveness in the first hours after cardiac surgery. We also found that the VTI-LVOT variability index has high specificity and a high positive likelihood ratio compared with the gold standard using a cut-off point of ≥ 12%.

**Conclusions:**

The VTI-LVOT variability index is a valuable tool for determining fluid responsiveness during the first 6 postoperative hours in patients undergoing cardiac surgery.

## Introduction

### Background

A current pillar in the treatment of patients in a critical condition, including patients who have undergone cardiac surgery, is the administration of intravenous fluids for initial resuscitation. While it is true that this is considered the basis for the management of shock, volume overload has been associated with longer mechanical ventilation time, kidney injury and even mortality [1, 2].

Various studies have shown a better prognosis in critically ill patients who receive more conservative fluid resuscitation [3, 4]. This benefit extends to those who have undergone cardiac surgery, in whom restrictive fluid therapy has been associated with less time on mechanical ventilation, a shorter stay in intensive care, the reduced incidence of acute kidney injury and improved mortality [5]. Thus, the potential benefits of volume expansion, and the consequent increase in cardiac output/oxygen delivery, must be balanced against the risk of aggravating pulmonary and tissue oedema [6].

Variation in stroke volume and its surrogates, induced by mechanical ventilation, were the first methods designed for the dynamic evaluation of fluid responsiveness. This is because during mechanical ventilation with positive pressure, pulmonary inflation reduces the preload and increases the afterload of the right ventricle. When this pressure is transmitted to the left side of the heart, the preload is also decreased at that level. When these variations induced by positive pressure reach cut-off points, both ventricles are preload-dependent [7–10].

It was established in 2000 that pulse pressure variation (PPV), as a surrogate for stroke volume variability, can be used to predict the cardiac output response to volume expansion [11]. This has been confirmed in multiple studies [12], and in a meta-analysis of 22 studies representing 807 patients. That meta-analysis reported a sensitivity of 88% for predicting fluid responsiveness and a specificity of 89%, with an average of 12% as a predictor value [13]; it also showed that the ranges of < 9% and > 13% predict non-response and fluid responsiveness, respectively. Results between these values are a ‘grey area’, within which, physicians cannot use this index to determine the need for fluid therapy. In such cases, a passive leg-raising test, end-expiratory occlusion test or ‘minifluid’ challenge may be a valuable alternative [14–16]. PPV has been used in other studies to evaluate the predictive value of different methods. For example, Hilbert et al. [17] compared the effectiveness of the dilation index of the carotid artery diameter with PPV and reported good results (r = 0.56).

Many other methods have been developed to assess stroke volume variation during mechanical ventilation. However, one limitation of such indices is the need for impractical and/or infeasible invasive devices. Thus, there is an increasing need for less invasive approaches [18]. These may include bedside ultrasound measurements such as variation in inferior vena cava dimensions and Doppler measurements of the left ventricular outflow tract (LVOT) as a stroke volume surrogate.

Most studies evaluating methods for fluid responsiveness prediction define this response as an increase in cardiac output or stroke volume after a fluid challenge, which requires invasive cardiac output measurements and fluid administration techniques that may be deleterious for the patient.

Echocardiography is currently used to measure peripheral haemodynamic indicators such as variability of the inferior vena cava, carotid flow and radial artery peak flow, with good predictive value. There is convincing evidence for the effectiveness of echocardiography in detecting vena cava collapse for predicting fluid responsiveness [19, 20]. However, various factors, such as ventilator parameters (tidal volume < 8 mL/kg), spontaneous breathing, right heart failure or intra-abdominal hypertension, can influence the sensitivity of this method; these factors mainly affect the predictive value of vena cava variability [21] and limit its widespread use.

Due to these limitations, a method that has become more popular as a surrogate for the measurement of stroke volume in critically ill patients is the velocity–time integral of the left ventricular outflow tract (VTI-LVOT), as the area of this tract remains relatively constant during the respiratory cycle. Importantly, it is less affected by vascular compliance and intra-abdominal pressure compared with other methods. In 2007, Lamia et al. [22] reported the effectiveness of measuring the increase in VTI-LVOT after passive leg raising in patients with spontaneous breathing, indicating that a cut-off of 12.5% was related to an increase in stroke volume of 15% with a sensitivity of 77% and a specificity of 100%. Other studies have confirmed these findings using the increase in VTI-LVOT after passive leg raising as a method to simulate a volume load [23, 24].

Most studies in which VTI-LVOT changes have been evaluated have correlated its modification after administration of a volume load. The only study to date that has focused on determining its value as a predictor of fluid responsiveness was carried out by Wang et al. [25]. In that study of patients diagnosed with sepsis, VTI-LVOT was measured prior to the volume challenge and compared with PPV after rapid volume loading. The authors found a good diagnostic performance (area under the curve = 0.956) with a sensitivity of 87.5% and a specificity of 95% to predict response to a fluid load using a VTI-LVOT cut-off of 15%.

Another study in patients with sepsis showed that VTI-LVOT peak velocity variability with a cut-off of ≥ 12% had a good positive predictive value (91%) for fluid responsiveness, defined as an increase in cardiac index ≥ 15% after volume loading, with a negative predictive value of 100% [26].

### Investigation goals

The goal herein was to determine the efficacy of ultrasound measurement of VTI-LVOT variability as a predictor of response to volume in patients undergoing cardiac surgery.

## Methods

This cross-sectional study investigated 50 consecutive adult patients who were admitted to the critical care unit at the Instituto Nacional de Cardiología Ignacio Chávez from 1 March to 30 October 2022, following cardiac surgery, in whom ultrasonographic evaluation was conducted upon arrival in postoperative critical care and in whom it was possible to obtain an adequate visualization of the five-chamber view. Patients with an open chest after surgery were excluded. For the ultrasonographic evaluation, the operator-obtained images were generated using a phased array sector probe at 2–3 MHz, from the patient’s right or left side, with sonographic equipment including the following modes: M-mode, 2D-mode, colour Doppler, pulsed wave Doppler, continuous wave Doppler and tissue Doppler. The use of sonographic equipment with advanced software technology was not necessary. The five-chamber view was obtained according to the guidelines of the American Society of Echocardiography for performing comprehensive transthoracic echocardiographic examinations in adults [27]. PPV was measured using a radial arterial catheter connected to an IntelliVue MX550 monitor (Philips Healthcare), with the transducer positioned at the level of the right atrium or the midaxillary line. The measuring system was also zeroed to obtain accurate data.

To determine the fluid responsiveness through the VTI-LVOT variability index, we used the following formula:

$$\left( {{\text{VTI}} - {\text{LVOT maximum}}{-}{\text{VTI}} - {\text{LVOT minimum}}} \right)/\left( {{\text{VTI}} - {\text{LVOT maximum}} + {\text{VTI}} - {\text{LVOT minimum/2}}} \right) \times {1}00 \, ( \ge {12}\% )$$

All patients were intubated and mechanically ventilated at a tidal volume of 8 mL/kg, without spontaneous inspiratory effort and without arrhythmias during measurements, as recommended for an adequate fluid responsiveness evaluation [28]. None of these patients had acute respiratory distress syndrome or another lung disease. Furthermore, their driving pressure and plateau pressure were within the range for protective lung ventilation. All included patients had normal right ventricular function parameters as evaluated by echocardiography, including tricuspid annular plane systolic excursion > 17 mm, tricuspid annular systolic velocity with tissue Doppler imaging > 9.5 cm/s and fractional area change > 35%.

Images were processed and analysed after acquisition. One physician (a critical care physician with training in critical care ultrasonography) acquired the images, after which, the images were processed and measured by three different physicians (clinical cardiologists with training in echocardiography, including DMS, EGC and ELD) using imaging software. We reduced bias by blinding the investigators who analysed, processed and measured the images. In all patients, as a regular measure of preload assessment, central venous pressure (CVP) was also measured invasively with a central venous catheter with the tip at the cavoatrial junction.

A PPV value ≥ 12 and a VTI-LVOT variability index of ≥ 12% were considered positive for fluid responsiveness.

### Statistical analysis

We performed the Shapiro–Wilk test of normality for continuous variables and report these as median and interquartile ranges because all were nonparametric. Comparisons of continuous variables were made using the Kruskal–Wallis test. We report categorical variables as frequencies and percentages and used the chi‐square or Fisher’s exact probability tests, as appropriate, to compare expected values. We used Pearson’s test to calculate correlation coefficients, with the following r value strength categories: 0.1–0.29 = small; 0.3–0.49 = medium and 0.50–1.0 = strong. We used 2 × 2 tables to calculate sensitivity, specificity, predictive values and likelihood ratios. All statistical analyses were performed using Stata v. 14, and p values < 0.05 were considered statistically significant.

## Results

### Demographic and surgical characteristics

Most of the patients (60% men, mean age: 55 years, age range: 42–65 years) were classified as New York Heart Association functional class II (62%). The most frequent comorbidities were arterial hypertension (42%), diabetes mellitus (20%) and heart failure (30%). Twenty-six per cent and 18% had a reduced or severely reduced left ventricular (LV) ejection fraction, respectively (Table 1). The most frequent surgery was aortic valve replacement (40%), followed by coronary artery bypass graft surgery (22%), with a mean extracorporeal circulation time of 153 min and aortic clamping for 101 min (Table 2).

**Table 1:** Baseline characteristics

Variable		n (%)
Women		20 (40)
Men		30 (60)
NYHA functional class	I	6 (12)
	II	31 (62)
	III	12 (24)
	IV	1 (2)
Prior myocardial infarction		5 (10)
Prior cardiac surgery		6 (12)
Diabetes		10 (20)
Chronic kidney disease		1 (2)
Heart failure		15 (30)
Hypertension		21 (42)
Normal EF		28 (56)
Reduced EF		13 (26)
Severely reduced EF		9 (18)

Variable	Median (IQR)
Age (years)	55.5 (42–65)

*NYHA* New York Heart Association, *EF* ejection fraction, *IQR* interquartile range

**Table 2:** Surgical characteristics

Variable		n (%)
Coronary artery bypass graft		11 (22)
Mitral valve replacement		3 (6)
Aortic valve replacement		20 (40)
Coronary artery bypass graft + aortic valve replacement		2 (4)
Aortic valve replacement + mitral valve replacement		3 (6)
Aortic surgery		1 (2)
Bentall procedure		5 (10)
Other		5 (10)
Surgical outcomes	Low output syndrome	10 (20)
	Mediastinal bleeding	8 (16)
	Vasoplegic syndrome	1 (2)

Variable	Median (IQR)
Extracorporeal Circulation time (min)	153 (106–203)
Aortic clamping (min)	101 (77–124)

### Haemodynamic measurements

**Fig. 1:**
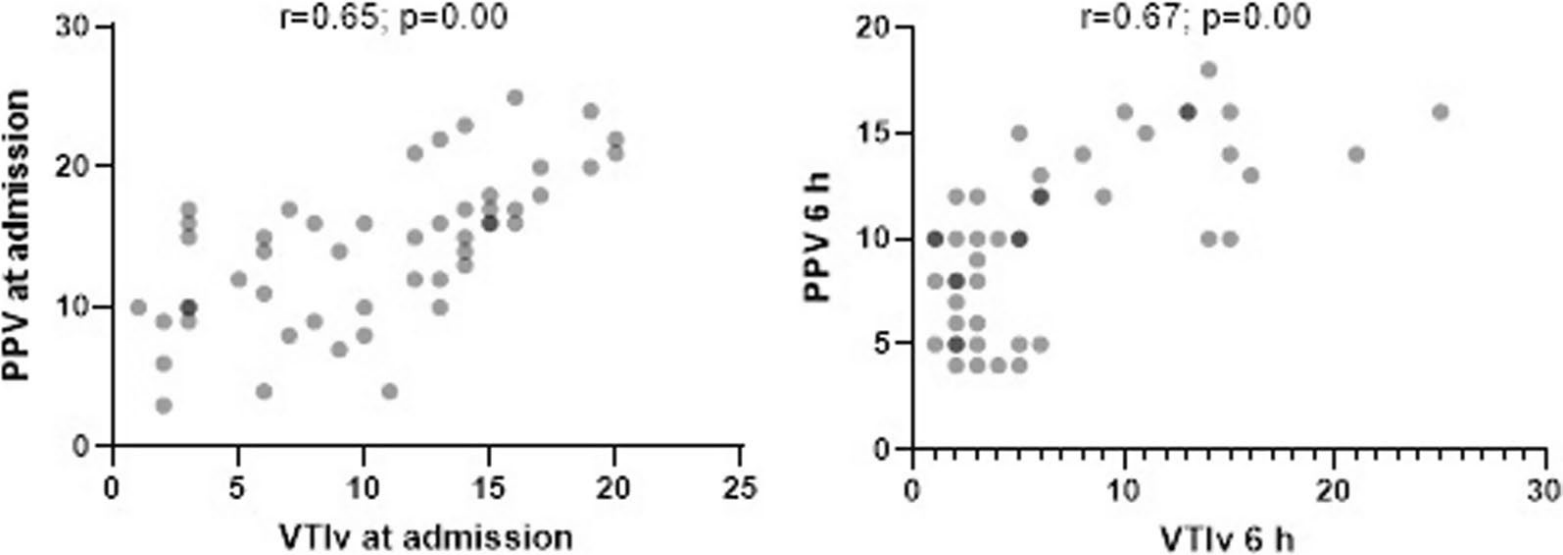
Correlation between the VTI-LVOT variability index and PPV at admission and 6 h. *PPV* pulse pressure variation, *VTI-LVOT* velocity–time integral of the left ventricular outflow tract

Among the overall sample, PPV obtained at admission was ≥ 12% in 68%, decreasing to 40% at 6 h, with 50% and 22% presenting a VTI-LVOT variability index ≥ 12% at admission and 6 h, respectively. Median CVP values at admission and 6 h were 8 mmHg and 10 mmHg, respectively (Table 3). Strong positive correlations between PPV and the VTI-LVOT variability index absolute value were seen at both admission (r = 0.65) and 6 h (r = 0.67) **(**Fig. 1**)**. When the diagnostic accuracy for fluid responsiveness of the VTI-LVOT variability index was evaluated compared with the PPV based on their cut-off points (≥ 12% for both), the sensitivity and specificity were 70% and 93% at admission and 44% and 92% at 6 h, respectively. Positive predictive values of 96% and 80% and negative predictive values of 60% and 71% were seen at admission and 6 h, respectively. Positive likelihood ratios of 11.3 and 6 were seen at admission and 6 h, respectively (Table 4).

**Table 3:** Hemodynamic measurements

Variable	Median (IQR)
PPV at admission (%)	15(10–17)
PPV at 6 h (%)	10(6–13)
CVP at admission (mmHg)	8(6–10)
CVP at 6 h (mmHg)	10(8–12)
VTI-LVOT maximun at admission (cm)	17(11–19)
VTI-LVOT minimun at admission (cm)	12(7–15)
VTI-LVOT maximun at 6 h (cm)	16(14–19)
VTI-LVOT minimun at 6 h (cm)	14(10–15)
VTI-LVOT variability index at admission (%)	11.5(6–15)
VTI-LVOT variability index at 6 h (%)	5(2–10)

Variable	N (%)
PPV at admission ≥ 12%	34 (68)
PPV at 6 h ≥ 12%	18 (40)
VTI-LVOT variability index at admission ≥ 12%	25 (50)
VTI-LVOT variability index at 6 h ≥ 12%	10 (22.2)

*PPV* pulse pressure variation, *CVP* central venous pressure, *VTI-LVOT* velocity–time integral of the left ventricular outflow tract

**Table 4:** Diagnostic accuracy of VTI-LVOT variability index to predict fluid responsiveness compared to pulse pressure variation

	VTI-LVOT variability index at admission Estimate 95% CI	VTI-LVOT variability index at 6 h Estimate 95% CI
Sensitivity	0.706 [0.538 to 0.832]	0.444 [0.246 to 0.663]
Specificity	0.938 [0.717 to 0.989]	0.926 [0.766 to 0.979]
PPV	0.96 [0.805 to 0.993]	0.8 [0.49 to 0.943]
NPV	0.6 [0.407 to 0.766]	0.714 [0.549 to 0.837]
LR +	11.387 [1.672 to 76.277]	6 [1.436 to 25.075]
LR −	0.313 [0.184 to 0.536]	0.6 [0.392 to 0.919]
Accuracy	78%	73.3%

*VTI-LVOT* velocity–time integral of the left ventricular outflow tract, *PPV* positive predictive value, *NPV* negative predictive value, *LR* likelihood ratio

## Discussion

There is evidence of the deleterious effect of excessive fluid resuscitation in critically ill patients, which translates into a longer stay in the intensive care unit, a higher incidence of kidney injury and increased mortality. Nevertheless, intravenous fluid administration continues to be the standard of care in the treatment of patients in shock, including those in the postoperative period of cardiac surgery.

Due to this shortcoming, efforts have been made over the years to find a balance between the risks and benefits of excessive fluid administration, leading to the development of methods that allow evaluation of the patient and whether they will have an adequate response to fluid administration, many techniques for which are invasive.

For this reason, evaluation at the bedside with ultrasound has now become relevant, with increasing evidence of its usefulness for blood volume analyses in critically ill patients. Most of the relevant studies have used fluid challenges that are often impractical in the daily clinical context. This has motivated the study of methods that allow minimally invasive evaluation, without the need for intervention and with good efficacy for determining whether the patient benefits from volume administration. These studies have begun in a few patients in the context of sepsis, and it is important to extend the spectrum of patients to those with cardiac pathology, and specifically following cardiac surgery. The Venous EXcess UltraSound score evaluates and scores the severity of venous congestion, assessing variations in hepatic, portal venous and renal venous waveforms, and correlating with an increased risk of acute kidney injury, to guide management of volume overload and limit fluid resuscitation efforts [29].

Herein, we show the usefulness of the VTI-LVOT variability index, a rapid, readily available and reproducible bedside echocardiographic measure for evaluating volume response during the early postoperative period of cardiac surgery. Measurement accuracy is essential during this immediate postoperative period, when evaluation of fluid responsiveness is fundamental for adequate patient management. This is especially true in this patient population, because of frequent hypovolemia due to blood loss or increased vascular permeability by the extracorporeal circulation pump and the inflammatory response to surgery [5]. Our results suggest a good correlation between the VTI-LVOT variability index and PPV as a method to assess fluid responsiveness.

All patients were intubated and mechanically ventilated and were thus ideally situated (regarding tidal volume and absence of spontaneous inspiratory effort) for predicting fluid responsiveness. These conditions may have increased the accuracy of our measurements, as invasive mechanical ventilation constitutes the optimal circumstances for these evaluations [30]. However, these conditions may not be achieved in all post-surgical patients, as our study was carried out during the first 6 post-surgical hours. Many patients can be adequately evaluated during this time, in which case, the VTI-LVOT variability index becomes a useful addition to other diagnostic methods [20, 21].

We routinely quantify CVP, not as a parameter of fluid responsiveness (as it depends on both cardiac and venous return function and does not help determine volume status reliably), as has already been extensively studied, but because CVP values > 12 mmHg are generally considered a limit for making additional efforts to replace fluids and limit volume overload. Herein, the 2 mmHg increase in CVP at 6 h coincides with the decrease in PPV and the VTI-LVOT variability index.

Variations in flow through the aorta measured by ultrasound have been found to be useful, specifically in patients in septic shock with mechanical ventilation and preserved ventricular function [27, 28]. Herein, a strong positive correlation was found between the VTI-LVOT variability index and the most effective non-invasive volume response study method, the PPV [14].

The high specificity shown herein at both admission and after 6 h is useful for ruling out the presence of fluid responsiveness. Furthermore, the high predictive positive value at admission may help determine whether those with a VTI-LVOT variability index ≥ 12% will indeed have a high probability to be fluid responsive, as confirmed by the positive likelihood ratio of 11.3. It is important to note that the decrease in diagnostic accuracy at 6 h is consistent with the dynamic process of fluid resuscitation, in that for the first 6 h, most patients are replete in intravascular volume. This was supported herein by the decrease in individuals responding to volume in the first 6 h compared with at admission.

During mechanical ventilation in the presence of LV dysfunction, the increase in pleural pressure that facilitates LV ejection (i.e., afterload reduction) is more pronounced. Thus, the effect of squeezing the pulmonary blood volume during early inspiration on the LV ejection is amplified, so a high PPV may be due to this variation, which would result in a false-positive PPV. However, few studies have addressed the issue of fluid responsiveness in patients with congestive heart failure, probably because fluid administration is rarely indicated in such patients. In some studies involving patients with LV dysfunction who are in sinus rhythm, PPV (or stroke volume variation) has been shown to predict fluid responsiveness with acceptable accuracy [20, 31].

### Study limitations

This study was carried out in a single hospital centre and must be replicated in other centres to assess the reproducibility of our results. Because our sample was relatively small, it will be necessary to evaluate a larger population to achieve better statistical power. Unlike other studies in which response to volume was evaluated with invasive ‘gold standard’ methods, we used a non-invasive method with the best diagnostic performance, which could degrade the reliability. Finally, it will be necessary in the future to consider both interobserver variability across centres and the need for personnel trained in these techniques.

## Conclusions

We found a strong positive correlation and adequate diagnostic accuracy between the VTI-LVOT variability index and PPV to predict fluid responsiveness in patients who underwent cardiac surgery.

## Data Availability

The datasets used and/or analysed during the current study are available from the corresponding author on reasonable request.
